# Less Frequent and Less Severe Flu-Like Syndrome in Interferon Beta-1a Treated Multiple Sclerosis Patients with at Least One Allele Bearing the G>C Polymorphism at Position -174 of the IL-6 Promoter Gene

**DOI:** 10.1371/journal.pone.0135441

**Published:** 2015-08-18

**Authors:** Diego Bertoli, Federico Serana, Alessandra Sottini, Cinzia Cordioli, Davide Maimone, Maria Pia Amato, Diego Centonze, Ciro Florio, Elisa Puma, Ruggero Capra, Luisa Imberti

**Affiliations:** 1 CREA, Diagnostics Department, Spedali Civili of Brescia, Brescia, Italy; 2 Multiple Sclerosis Center, Spedali Civili of Brescia, Presidio di Montichiari, Brescia, Italy; 3 Department of Neurology, Garibaldi Hospital, Catania, Italy; 4 Department of Neurology, University of Florence–Careggi Hospital, Florence, Italy; 5 Department of Neurosciences Tor Vergata University, S. Lucia Foundation IRCCS, Rome, Italy; 6 Department of Neurology, Ospedale Caldarelli, Napoli, Italy; 7 Biogen Italy, Medical Department, Milan, Italy; Washington University, UNITED STATES

## Abstract

One of the most common adverse event of interferon beta (IFNβ) therapy for multiple sclerosis is flu-like syndrome (FLS), which has been reportedly related to increased levels of cytokines such as interleukin 6 (IL-6) and tumor necrosis factor-alpha (TNF-α). Average cytokine levels can be affected by single nucleotide polymorphism in the gene promoter regions. To investigate whether IL-6 -174 G>C and TNF-α -376 G>A polymorphisms could be correlated to the incidence of FLS, and whether an anti-inflammatory/antipyretic therapy may influence FLS development, a prospective observational study was performed in 190 treatment naïve, multiple sclerosis patients who started IM IFNβ-1a 30mcg once weekly. The identification of IL-6 -174 G>C and TNF-α -376 G>A polymorphisms was achieved by performing an amplification-refractory mutation system. Serum IL-6 levels were measured using enzyme-linked immunosorbent assay in blood samples taken before therapy and then after the first and last IFNβ-1a injection of the follow-up. FLS-related symptoms were recorded by patients once per week during the first 12 weeks of therapy into a self-reported diary. We found that patients carrying at least one copy of the C allele at position -174 in the promoter of IL-6 gene produced lower levels of IL-6 and were less prone to develop FLS, which was also less severe. On the contrary, the polymorphism of TNF-α had no effect on FLS. Patients taking the first dose of anti-inflammatory/antipyretic therapy in the peri-injection period (within 1 hour) experienced a reduced FLS severity. In conclusion, the study of IL-6 -174 G>C polymorphism would allow the identification of patients lacking the C nucleotide on both alleles who are at risk of a more severe FLS, and may be addressed to a timely and stronger anti-inflammatory/antipyretic therapy for a more effective FLS prevention.

## Introduction

Interferon beta (IFNβ) is a well-established, first-line, disease-modifying therapy used for multiple sclerosis (MS) that has a demonstrated efficacy in relapsing remitting MS. It has a manageable tolerability profile, with no unexpected adverse events observed after over a 16-year follow-up period [1]. However, several patients experience systemic side effects after treatment initiation [2]. The most common adverse events are injection site reactions, headache, and flu-like syndrome (FLS). FLS, which include fever, sweating, muscle aches, and malaise, usually begins 3–6 hours after IFNβ injection and may last for 8 to 24 hours [3,4,5]. Its symptoms typically decline during the first 3 months of treatment, but may continue or recur in some patients [6]. Because some patients never tolerate the therapy, FLS can lead to an impaired treatment adherence and to early dropouts [7]. Analgesic/antipyretic drugs such as non-steroid anti-inflammatory drugs and oral corticosteroids are used to manage FLS [8], but the understanding of the reasons for FLS development after IFNβ may improve the management of these symptoms, so that all patients can fully benefit from the therapy.

FLS symptoms have been reportedly related to increased endogenous pyrogens such as interleukin 6 (IL-6) and tumor necrosis factor-alpha (TNF-α) [9]. For instance, IL-6 increases significantly in patients who developed FLS with fever during the first weeks of IFNβ treatment. These side effects can be ameliorated by steroids [10]. Accordingly, *in vitro* analysis of IL-6 production has been proposed for the identification of patients who are likely to develop fever at the beginning of IFNβ therapy [11]. TNF-α was also reported to be involved in the induction of fever, but with contrasting results [12,13,14,15,16]. Furthermore, high levels of TNF-α have been found in MS plaques and cerebrospinal fluid (CSF) [17], with CSF levels that correlated well with disability and the rate of neurological deterioration [18].

Cytokine expression can be influenced by single nucleotide polymorphisms (SNPs) in the promoter regions of their genes [19,20,21]. For instance, several polymorphisms have been described in the IL-6 promoter region. Among them, the single nucleotide change from G to C at position -174 (IL-6–174 G>C) has been found to suppress IL-6 transcription, and to result in lower plasma IL-6 levels [22]. Analogously, several TNF-α gene polymorphisms have been associated with gene transcription modifications [21], and that at position -376 (G>A), which was associated with susceptibility to MS [23], may also be involved in altered TNF-α levels.

However, while it has been recently demonstrated that IFNβ-1a significantly suppressed plasma IL-6 and TNF-α levels [24], no data were available on cytokine gene polymorphism as a possible cause of the differential modulation of these cytokines and/or FLS in MS patients. Therefore, the study’s primary end-point was to investigate whether IL-6 -174 G>C and TNF-α -376 G>A polymorphisms could be correlated to the incidence of FLS in MS patients who started IFNβ-1a IM therapy. The levels of IL-6 in their peripheral blood were measured by an enzyme-linked immunosorbent assay before therapy and then after the first and last IFNβ injections of the follow up, and FLS was defined according to a patient self-reported diary scoring the severity of FLS.

## Patients and Methods

### Patients

Overall, 190 treatment naïve patients with relapsing-remitting MS, diagnosed according to McDonald revised criteria [25], were enrolled in 28 Italian centers over a period of 23 months. They did not receive steroids for at least 1 month before starting the therapy with IM IFNβ-1a 30 mcg once weekly, which was given according to the Italian Medicines Agency rules.

Blood was collected just before the beginning of IFNβ-1a IM therapy, after the first injection, and then at 12 weeks of therapy. Samples were obtained about 12 hours after the injection because the IL-6 serum level increases 10 hours after IFNβ-1a injection and returns to the basal level 24 hours later [26]. Blood samples from 100 healthy subjects taken and stored by the central laboratory were chosen as age- and gender-matched healthy controls (HC).

#### Ethics Statement

The study was approved by the ethical committee of Spedali Civili of Brescia (resolution n.582 12-04-2010), and all patients signed a written informed consent.

#### FLS self-reporting

Four of the several possible FLS-related symptoms (fever, malaise, sweating, and muscle aches) had to be recorded by patients during the first 12 weeks of therapy into a self-reported diary once weekly (in concomitance with every IFNβ-1a administration). Patients were instructed to score the symptom severity on a scale ranging from 0 (absent) to 3 (important) (S1 Table). In the case of fever, the score was coded based on the body temperature (1 if >37°C and <38°C; 2 if >38°C and <39°C; 3 if >39°C). Patients were also required to report any anti-inflammatory/antipyretic (nonsteroidal anti-inflammatory drugs, acetaminophen, and corticosteroids) drugs consumed before and/or after each drug injection (which may have affect the scoring of the symptoms and, thus, the analysis).

### Laboratory evaluations

#### IL-6 determination in sera

Serum IL-6 levels were measured using a commercial quantitative sandwich enzyme-linked immunosorbent assay according to the kit procedure (Quantikine HS Human IL-6 kit; R & D Systems, Minneapolis, MN). The assay range was 0.156–10 pg/ml; the minimum detectable dose was 0.039 pg/ml and lower levels were considered undetectable.

#### Preparation of DNA and IL-6 and TNF-α single nucleotide polymorphism (SNP) analysis

The identification of IL-6 -174 G>C (rs1800795) and TNF-α -376 G>A (rs1800750) polymorphisms was achieved by performing an amplification-refractory mutation system PCR. Genomic DNA was extracted from peripheral blood using the QIAamp DNA Blood Midi Kit (Qiagen GmbH, Hilden, Germany). Two different reaction solutions were prepared, both with a final volume of 50 μl, which contained 5 μl of 10X Buffer II, 3 μl of MgCl_2_ 25mM, 5 μl of GeneAmp dNTP Blend mix (Life Technologies, Carlsbad, CA) 0,2 mM each, 0,2 μl of AmpliTaq Gold DNA Polymerase (Life Technologies, Carlsbad, CA) 5U/ μl, 10 pmol of IL-6 and TNF-α reverse primers and 25 ng of DNA. Ten pmol of IL-6–174 C and TNF-α -376 A forward primers were added to the first reaction, while 10 pmol of IL-6–174 G and TNF-α -376 G forward primers to the second one. The primer sequences are shown in Table 1.

**Table 1:** Primers for ARMS-PCR and sequencing analysis of IL-6 and TNF-α genes.

	Target	Primer Sequence
**ARMS-PCR**	IL-6–174 C forward	5'-CTGCACTTTTCCCCCTAGTTGTGTCTTGCC-3'
	IL-6–174 G forward	5'-TCCCCCTAGTTGTGTCTTGCG-3'
	IL-6 reverse	5'-TGAGGGTGGGGCCAGAGC-3'
	TNF-α -376 A forward	5'-CTATCTTTTTCCTGCATCCTGTCTGGAAA-3'
	TNF-α -376 G forward	5'-TTCCTGCATCCTGTCTGGAAG-3'
	TNF-α reverse	5'-GCCACTGACTGATTTGTGTGTAGG-3'
**Sequencing**	IL-6 forward	5'-TTGTCAAGACATGCCAAAGTGCTGA-3'
	IL-6 reverse	5'-TGAGGGTGGGGCCAGAGC-3'
	TNF-α forward	5'-CAGCTCCTTCTCCCCGCAG-3'
	TNF-α reverse	5'-GCCACTGACTGATTTGTGTGTAGG-3'

Amplification parameters were: a cycle at 95°C for 7 min; 35 cycles at 95°C for 30 sec, 58°C for 30sec, and 72°C for 30 sec); a cycle at 72°C for 10 min. PCR products were visualized by electrophoresis on 2.5% agarose gel. The estimated sizes of PCR products for IL-6 -174 C and TNF-α -376 A alleles were 104 and 144 bp, respectively; the expected band sizes for IL-6 -174 G and TNF-α -376 G alleles were 95 and 136 bp, respectively. In each experiment, a known individual DNA heterozygous for both loci was included as positive control to ensure amplification of both alleles for both targets. The reaction solution without DNA served as negative control.

In order to confirm the results obtained by this method, IL-6 and TNF-α genotypes of some subjects were also assessed by direct sequencing analysis. Amplification products to be sequenced were obtained using the primers reported in Table 1 with the same PCR conditions used for the amplification-refractory mutation system PCR.

### Statistical analysis

Because the level of IL-6 was not normally distributed, its values were log-transformed. The measures of IL-6 over time in the genotype subgroups were compared using ANOVA based on mixed-model regression with a random intercept. Univariate associations between categorical variables were assessed by the Fisher’s exact test. These analyses were performed only the subjects for whom results of genotyping and IL-6 determination were both available. When dichotomous categorical variables were used to assess the presence of an event during the 12 weeks of follow-up (e.g. post-injection FLS), the differences in the frequency of the event between the genotype subgroups were evaluated by mixed effects logistic regression, which also allowed us to adjust results for confounding factors or covariates. Stepwise selection and a likelihood test were used to choose the final model. The mixed effects models, both linear and logistic, were utilized also because they have the advantage of not requiring a balanced design, thus allowing the use of all observations during the follow-up, disregarding the issue of missing data. On the contrary, the area under the curve (AUC) for FLS severity score was calculated considering only patients in whom no missing information was present in the diary (n = 121), and comparison of its median values between subgroups was made by the Mann-Whitney test. Significance threshold was set at the p value of 0.05.

## Results

### Study population

The median age of the 190 enrolled patients was 38 years (range 18–63), 69.3% were female. Median MS disease duration was 3 months, with a mean Expanded Status Disability Score equal to 1.53.

The laboratory tests were performed on 159 patients because 3 were lost to follow-up and 28 discontinued the therapy because of adverse events (4), investigator decisions (6), consent withdrawal (8), and worsening of disease (10). In 9 patients, only serum samples were obtained, and thus genotyping could not be performed; in 3 patients IL-6 levels could not be quantified for technical problems; therefore, a matching genotype-cytokine information was available in 147 patients, who are the subjects analyze189/d in the study.

### IL-6 and TNF-α genotyping and circulating IL-6 levels

Genotype distribution and allelic frequencies of the IL-6 -174 G>C and TNF-α -376 G>A polymorphisms did not differ between MS patients and HC (Table 2).

**Table 2:** Genotype distribution and allelic frequencies of the IL-6–174 G>C and TNF-α -376 G>A polymorphism in MS patients and HC.

IL-6			TNF-α		
	Patients	HC^a^		Patients	HC
	(n = 147)	(n = 107)		(n = 147)	(n = 107)
**Genotype**	**%**	**%**	**Genotype**	**%**	**%**
G/G	55.8	51.4	G/G	92.5	95.3
C/G	36.7	37.4	A/G	6.8	4.7
C/C	7.5	11.2	A/A	0.7	0
**Allele**	**%**	**%**	**Allele**	**%**	**%**
G	74.1	70.1	G	95.6	97.7
C	25.9	29.9	A	4.4	2.3

^**a**^healthy controls

**Fig 1:**
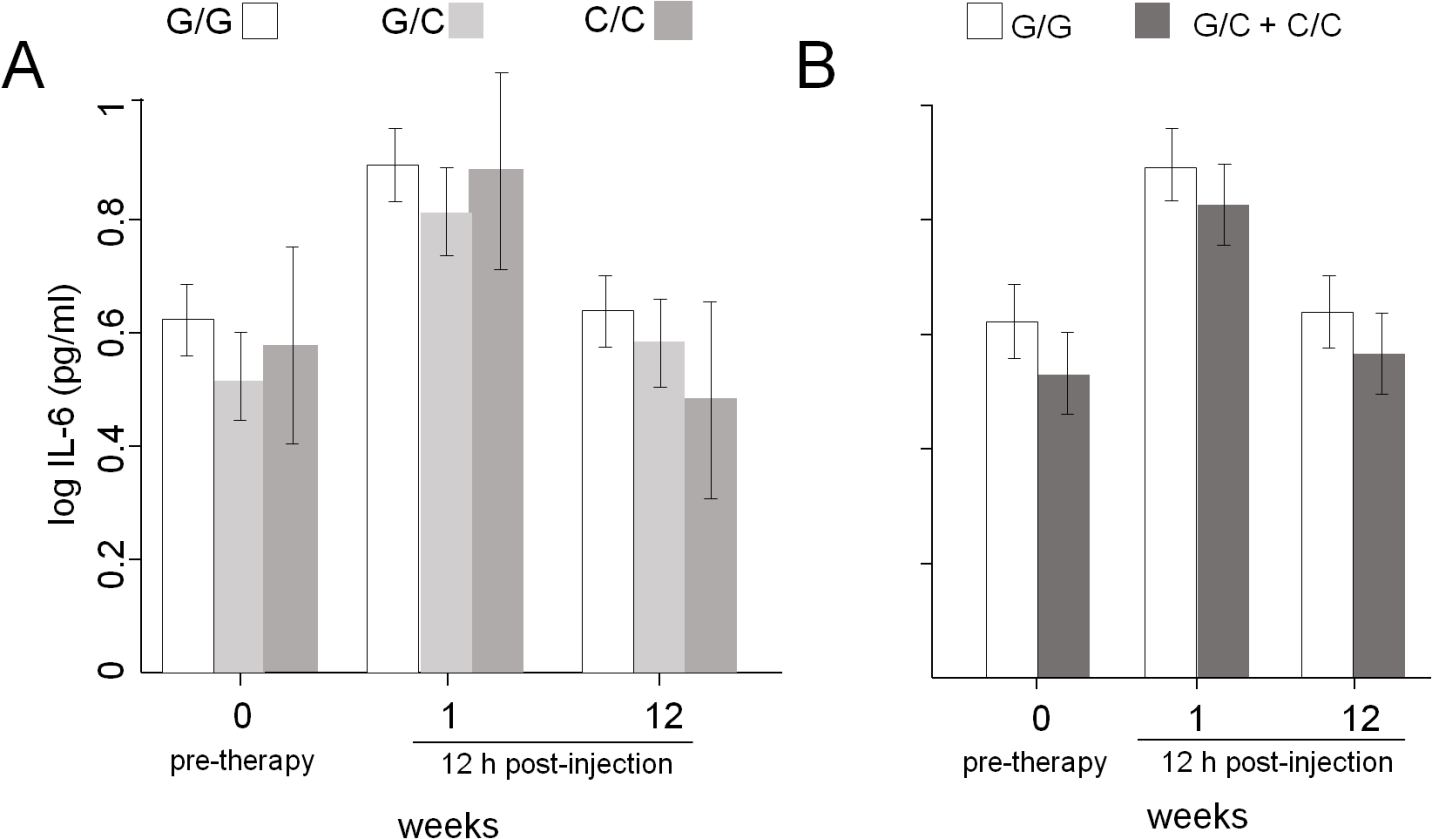
Average IL-6 levels in MS patients. (A) Average IL-6 levels in patients with the three indicated IL-6 genotypes. (B) Average IL-6 levels in patients bearing at least one allele with a C at position -174 of IL-6 gene and in those bearing only the allele with the G at the same position. 0 = samples collected just before the beginning of IFNβ-1a therapy; 1 = samples collected at about 12 hours after the first injection; 12 = samples collected at about 12 hours (h) from the injection performed at 12 weeks of therapy.

Before treatment, serum IL-6 levels were higher in MS patients than HC (mean 4.21 vs 1.41 pg/ml, p<0.001), and thereafter significantly changed during the follow up (ANOVA main effect of time: p<0.001; interaction term non-significant. Fig 1A), because they increased immediately after the first 12 hours of IFNβ-1a administration (p<0.001), and only slightly decreased in the 12 subsequent weeks (p<0.001). There were no significant differences between the three IL-6 genotype subgroups in the average IL-6 levels neither in MS patients (ANOVA main effect of genotype: p = 0.10; Fig 1A) nor in HC (S1 Fig), and there were no differences in the pattern of IL-6 decrease (interaction term non-significant). However, a significant difference was observed only in MS patients if they were reclassified in order to make a comparison between those bearing or not bearing the C allele of the IL-6 gene. Indeed, patients with the C allele had, on average, significantly lower IL-6 levels (ANOVA main effect of genotype: p = 0.03; Fig 1B), whereas the pattern of IL-6 decrease was still unchanged (interaction term non-significant). In contrast, no differences in IL-6 levels were found between patient groups based on the TNF-α polymorphic alleles (data not shown).

### FLS frequency and severity

**Fig 2:**
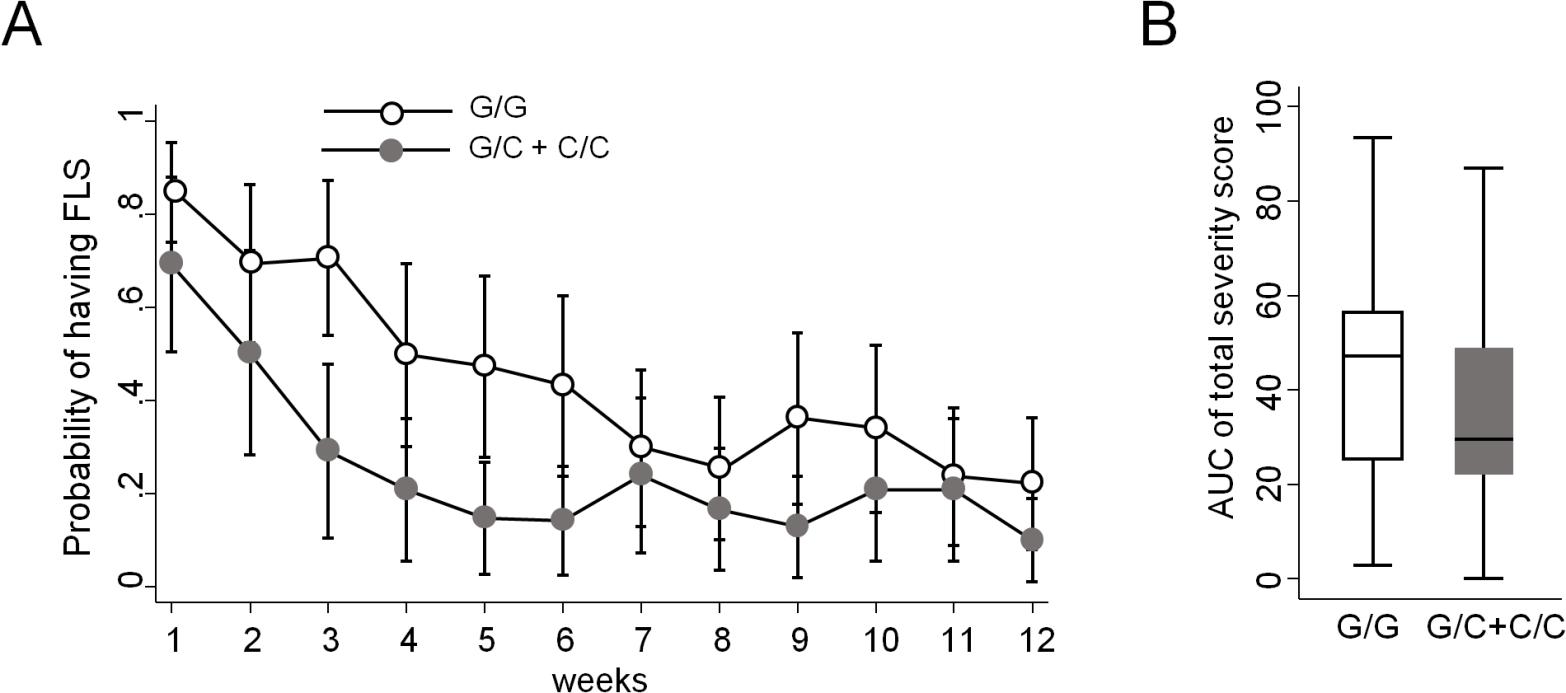
FLS probability and severity. (A) Probability to develop FLS calculated during the follow-up of 12 weeks in patients bearing at least one allele with a C at position -174 of IL-6 gene and in those bearing only the allele with the G at the same position. (B) Area under the curve (AUC), calculated as the total symptoms scores that were self-reported after each injection in patients bearing at least one allele with a C at position -174 of IL-6 gene and in those bearing only the G allele at the same position.

FLS was defined according to the number and intensity of the self-reported symptoms recorded in the patient diary (S2 Fig). FLS was considered as present after any one of the weekly IFN-β injections, when the patient self-reported at least two symptoms with a score 2 or 3 (except for fever, for which the score 1 was considered sufficient). Based on this definition, the weekly presence of FLS in a given patient varied during time, but an average trend towards a decrease in FLS prevalence during the follow-up was observed, with lower values in patients having at least one allele with the polymorphic nucleotide C at position -174 of IL-6 gene (65 out of 147 patients; Table 3). In order to calculate the statistical significance of this trend properly (i.e. keeping into account the repeated observations over time), mixed-model logistic regression was employed. This analysis confirmed that the probability to develop FLS decreased significantly with time, dropping below 50% already after the first month, and that there are differences between patients bearing vs those not bearing at least one copy of the polymorphic C allele of IL-6 (OR: 0.37, CI: 0.16‒0.84). Indeed, the presence of the allele C reduces, on average, the probability of having FLS by 19.3% (CI: 4.4%‒34.3%) (Fig 2A), and this effect does not change over time (interaction term non-significant). On the contrary, the polymorphism of TNF-α did not have any influence on FLS (not shown).

**Table 3:** Average weekly FLS prevalence in patients divided based on the IL-6 genotypes or on the presence of the C allele.

week	G/G	C/G	C/C	C/C+C/G
	(n = 82)	(n = 54)	(n = 11)	(n = 65)
1	0.72	0.64	0.40	0.60
2	0.61	0.51	0.40	0.49
3	0.63	0.40	0.30	0.38
4	0.49	0.31	0.36	0.32
5	0.48	0.27	0.30	0.27
6	0.45	0.27	0.30	0.27
7	0.38	0.34	0.36	0.34
8	0.35	0.31	0.18	0.29
9	0.42	0.25	0.27	0.25
10	0.39	0.31	0.36	0.32
11	0.34	0.31	0.36	0.32
12	0.32	0.24	0.18	0.23

The diary also allowed us to quantify FLS severity, which was initially evaluated as the area under the curve of the total symptoms scores that were self-reported after each injection. This analysis demonstrated a significantly lower FLS severity in patients bearing the IL-6 allele C vs patients bearing only the G allele (p = 0.04, Fig 2B). A finer analysis assessing the change in the average weekly FLS scores over time (i.e. evaluated by mixed-model regression after each injection, including patients with missing data at some time-points) revealed a similar trend in the difference between the average scores in the two subgroups of patients, together with a significant decrease in FLS severity score over time (S3 Fig).

### Effects of symptomatic therapy on FLS

**Fig 3:**
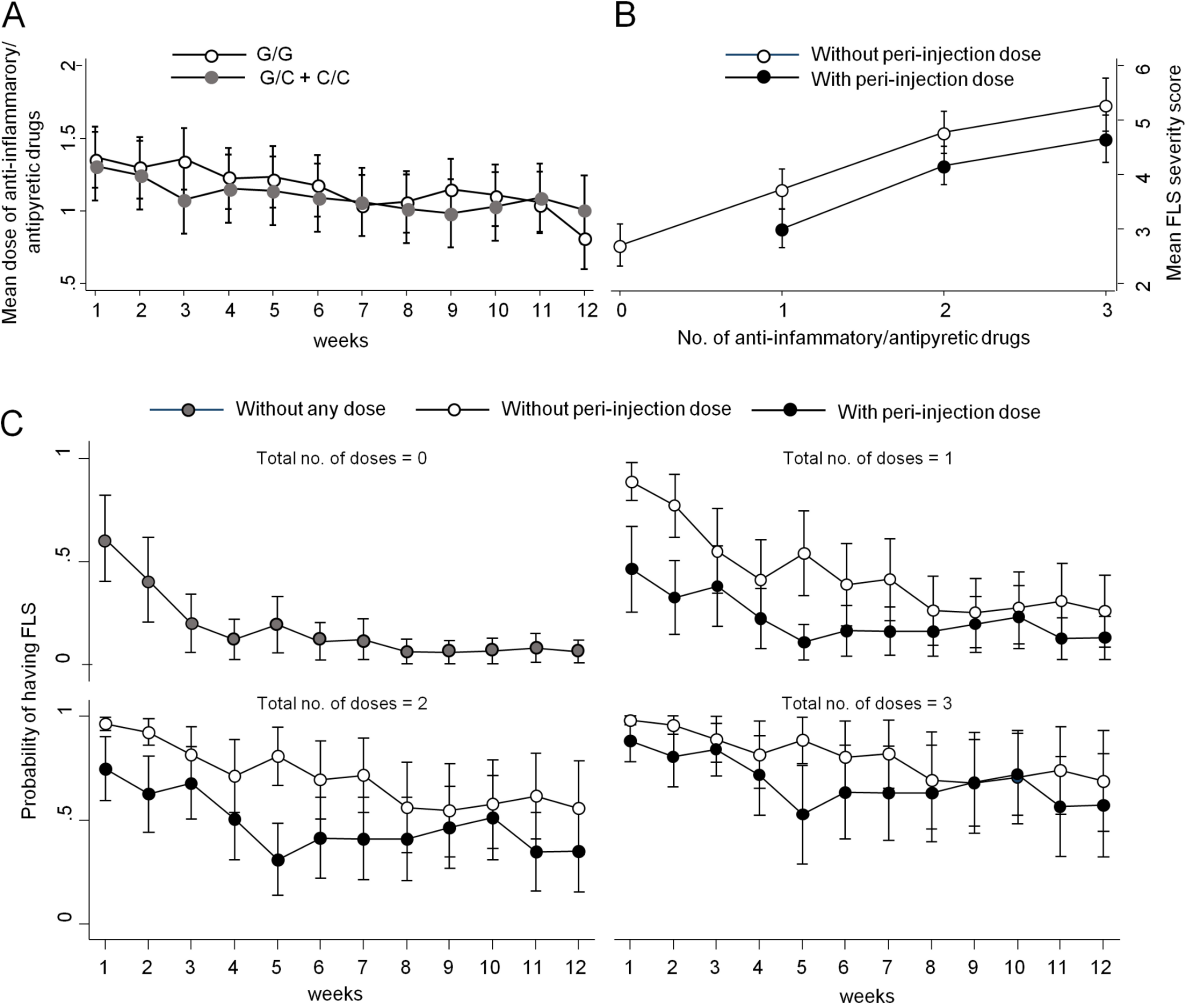
Consumption of anti-inflammatory/antipyretic drugs and its effects on FLS. (A) Comparison of mean number (no.) of anti-inflammatory/antipyretic drugs taken during the follow-up by patients bearing at least one C allele at position -174 of IL-6 gene vs. those bearing two G alleles at the same position. (B) Comparison of mean FLS severity score in patients taking the first dose of anti-inflammatory/antipyretic drugs in the peri-injection period vs. those that assumed the first dose hours later. (C) Effect of the peri-injection dose of antipyretic/anti-inflammatory drug on the probability of developing FLS in patients taking different total doses of antipyretic/anti-inflammatory drugs.

The study was performed in the conditions of common clinical practice and, therefore, patients were instructed to assume anti-inflammatory/antipyretic drugs before or after each injection, depending on the onset and intensity of FLS symptoms. Because this symptomatic, supportive therapy could potentially affect the reporting and scoring of FLS symptoms, patients were asked to record in the diary not only the time of each IFNβ injection, but also the exact time when the other drugs were taken. Based on this information, we calculated the total number of doses of anti-inflammatory/antipyretic drugs received per injection (mean dose: 1.1 dose/injection; range: 0–3) and per patient (mean total doses in 12 weeks of follow-up: 13.2; range: 0–36). A decreased use of anti-inflammatory/antipyretic drugs was observed over time, with a pattern roughly paralleling that of FLS severity, without any significant differences between patients bearing the different genotypes or alleles (Fig 3A; ANOVA main effect of time: p<0.001).

The first dose of anti-inflammatory/antipyretic therapy was preventively taken before or no later than 1 hour after the injection (defined as peri-injection period) in an average of 49% of total injections, and, therefore, in the other 51% of injections it was assumed hours later. After adjusting for this factor, we found that the post-treatment IL-6 levels were affected neither by the consumption of a peri-injection dose nor by the total dose of anti-inflammatory/antipyretic drug (data not shown). In spite of this, multivariable regression analysis demonstrated that when the severity of FLS was higher, patients assumed a higher number of doses, and that those who consumed the first dose in the peri-injection period experienced a FLS of lower intensity (Fig 3B; p<0.001). Therefore, both the total number of doses of anti-inflammatory/antipyretic drugs and the peri-injection doses were included as covariates in a new multivariable model demonstrating that the adjusted probability to develop FLS was affected not only by IL-6 genotype, but also by the number of anti-inflammatory/antipyretic doses taken, and by time of first intake (S2 Table). Indeed, when the same number of anti-inflammatory/antipyretic doses was taken, the presence of at least one polymorphic allele C of IL-6 gene reduced FLS probability in comparison to patients not bearing the C allele (OR: 0.36; 95% CI: 0.15‒0.85; p = 0.018). In addition, the intake of a peri-injection dose of anti-inflammatory/antipyretic drug further reduces the probability of experiencing FLS symptoms, albeit this effect is more apparent for a total of 1 and 2 doses, and during the first months of therapy with IFNβ, while it later decreases following the waning of FLS (Fig 3C).

## Discussion

This study’s aim was to find factors that could explain the variable presentation of FLS in distinct patients, and may be useful to guide the choice of the most appropriate first-line treatment of MS in cases when a high risk of FLS may be considered inappropriate or not tolerated.

Indeed, after more than 20 years of IFNβ use, the reasons underlying the variability of FLS presentation are not fully understood. Even if the onset of FLS had been linked to the blood levels of cytokines, such as IL-6 and TNF-α [16], and their levels appear to be related to SNPs in promoter regions, no study had previously assessed whether these genetic features allow a prediction of the frequency or extent of FLS in individual patients. To this end, we designed a prospective, observational study, to evaluate FLS in relation to the IL-6 and TNF-α SNPs in patients who started IFNβ-1a IM therapy and were followed up in the conditions of normal clinical practice. IL-6 levels were measured before and after the first IFNβ injection, and then after 12 weeks.

A strength of the present study was the self-reported questionnaire, in the form of a diary filled by participating patients after each injection for 12 weeks. To define FLS adequately, among the wide spectrum of symptoms we chose four (fever, malaise, sweating, and muscle aches) that could be easily, precisely, and unequivocally linked to the biological action of IFNβ. Of note, all were used also by Reess et al [27], and three of them by Rio et al [28], in two prospective studies where a similar patient-filled diary was also used. Although very common during FLS, headache was not included in this study because it is frequently related to other etiological factors, and its presence may be redundant and potentially confounding.

The frequency and, to some extent, the severity of FLS were found to be correlated to a polymorphic site at position -174 of the IL-6 gene, because they were reduced by the presence of at least one allele bearing a G>C substitution (occurring in nearly half of the patients). This is particularly meaningful because this polymorphic allele reduced the average levels of IL-6, which, in turn, is considered one of the main determinants of FLS [16]. Indeed, our data show that the decrease of post-injection IL-6 levels goes in parallel with the decrease in FLS frequency and severity over the course of treatment. Already after the first month of treatment, the probability of having FLS, as it was defined in the present study (at least two out of four symptoms scoring > = 2, or one symptom scoring > = 2 plus any fever), dropped to less than 50%. A similar time-dependent decrease in FLS prevalence had been previously reported [27].

FLS severity, defined according to the self-reported symptoms scores, also decreased in a similar fashion over time. Despite this time-trend, an extensive use of post-injection anti-inflammatory/antipyretic drugs was reported in the majority of patients throughout the follow up. Indeed, the total amount of anti-inflammatory/antipyretic drugs appeared correlated to the severity of the symptoms; i.e. at any given point during the follow-up, patients perceiving a more severe FLS assumed more anti-inflammatory/antipyretic drugs. An exception to this association is the first dose of anti-inflammatory/antipyretic drugs that many patients assumed preventively, before or immediately after IFNβ injection (i.e. before FLS onset). Indeed, after stratifying patients based on this behavior, the risk of developing FLS and its severity were found to be slightly lower in those assuming an anti-inflammatory/antipyretic drug in the peri-injection period, independently of the final number of doses and of the genotype, both of which continued to represent an important and independent determinant of FLS risk. In other words, our model suggest that both the differential post-IFNβ activation of the IL-6 system (due to -174 G>C polymorphism) and the variable use of anti-inflammatory/antipyretic drugs affect the frequency and severity of FLS, which, however, maintains a spontaneous tendency to decrease over time.

Despite studies showing that IL-6 levels can be lowered by a sustained administration of drugs of the nonsteroidal anti-inflammatory class [29] or steroids [10] we did not detect a direct effect of the anti-inflammatory/antipyretic drugs on the mean IL-6 levels, which were higher in MS patients than in HC as previously observed [30]. Therefore, the effect of the anti-inflammatory/antipyretic therapy on FLS in our patients is likely to be mediated by other mechanisms, such as downstream signals or interference with other cytokines, at least at the intermittent dosage used in our setting that may not be high enough to determine a detectable reduction of IL-6 levels.

For the correct interpretation of our results it should be kept into account that the prevalence of the IL-6 -174 G>C polymorphism varies between different populations [31]. In addition, in condition of systemic inflammation, the polymorphism effect on IL-6 levels can vary, depending on the underlying disease or condition [32,33,34,35], or pharmacological intervention (IFNβ vs. other treatment) [36]. Therefore, the impact of our results should be carefully weighed in the right context.

A possible limitation of this study is the method utilized to measure the dose of anti-inflammatory/antipyretic drugs consumed. Indeed, we oversimplified the concept of “dose”, disregarding the fact that one pill may contain a different amount of drug (e.g. acetaminophen 500 mg or 1000 mg) in distinct occasions/patients. Moreover, the use of anti-inflammatory/antipyretic drugs may have been reported in a heterogeneous way among different patients. The fact that FLS significantly decreases if IFNβ is injected in the morning [37] should not bias our results because the high majority of our patients performed the injection in the evening or during the night (and therefore we did not adjust the analysis for this covariate). Furthermore, the phenomenon of IFNβ bioactivity loss is not likely to bias our results because during the first 3-months of treatment only non-neutralizing antibodies are usually found, and nearly always against IFNβ-1b [38].

In conclusion, we demonstrated that IL-6 levels are lower in MS patients having at least one C allele at position -174 of IL-6 gene, which in turn determined a reduced probability of IFNβ-related FLS. Even if FLS incidence and severity tends to decrease spontaneously over the weeks, assuming anti-inflammatory/antipyretic drugs in the peri-injection period is of help in reducing FLS symptom severity. A recently published study demonstrated that a combination of easily learnt non-pharmacological techniques such as meditation, breathing practices, and a bizarre exposure to cold reduced the levels of IL-6 and TNF-α during experimentally induced FLS in healthy volunteers following lipopolysaccharide administration [39]. At least some of these procedures might in theory be helpful in reducing also the post-IFNβ FLS severity in MS patients. Therefore, the identification of patients lacking the C allele, who are at higher risk of FLS, could have important implications for IFNβ treated patients, who may be switched to stronger pharmacological as well as to alternative treatments for FLS management. This approach would likely enhance their overall compliance to IFNβ therapy.

## Supporting Information

S1 Dataset Dataset used for the analysis. (ZIP)

S1 Fig Average IL-6 levels in healthy controls. Average IL-6 levels in healthy controls divided according to the indicated IL-6 genotypes. No significant differences are present between subgroups bearing different genotypes.
(PDF)

S2 Fig Frequency distribution of FLS symptoms’ scores. Histograms showing the change in symptoms’ score distribution over the follow-up.
(PDF)

S3 Fig Average FLS severity scores in MS patients divided according to the indicated IL-6 genotypes. Graph represent the average FLS severity scores in MS patients divided according to the indicated IL-6 genotypes.
(PDF)

S1 Table Self-reported symptoms scoring as reported in patient diary. (PDF)

S2 Table Results of the multivariable repeated-measures logistic regression used to estimate the probability of having FLS after each weekly injection. (PDF)
