# Supplementary figures and images for: Less Frequent and Less Severe Flu-Like Syndrome in Interferon Beta-1a Treated Multiple Sclerosis Patients with at Least One Allele Bearing the G>C Polymorphism at Position -174 of the IL-6 Promoter Gene
Source: PLoS One. 2015 Aug 18;10(8):e0135441. doi: 10.1371/journal.pone.0135441 (PMC4540473; doi:10.1371/journal.pone.0135441)

S1 Figure

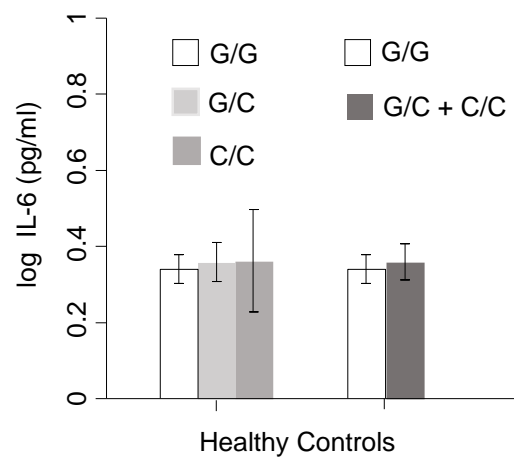

Supplement: S1 Fig — Average IL-6 levels in healthy controls divided according to the indicated IL-6 genotypes. No significant differences are present between subgroups bearing different genotypes. (PDF) [file pone.0135441.s002.pdf]

# S2 Figure

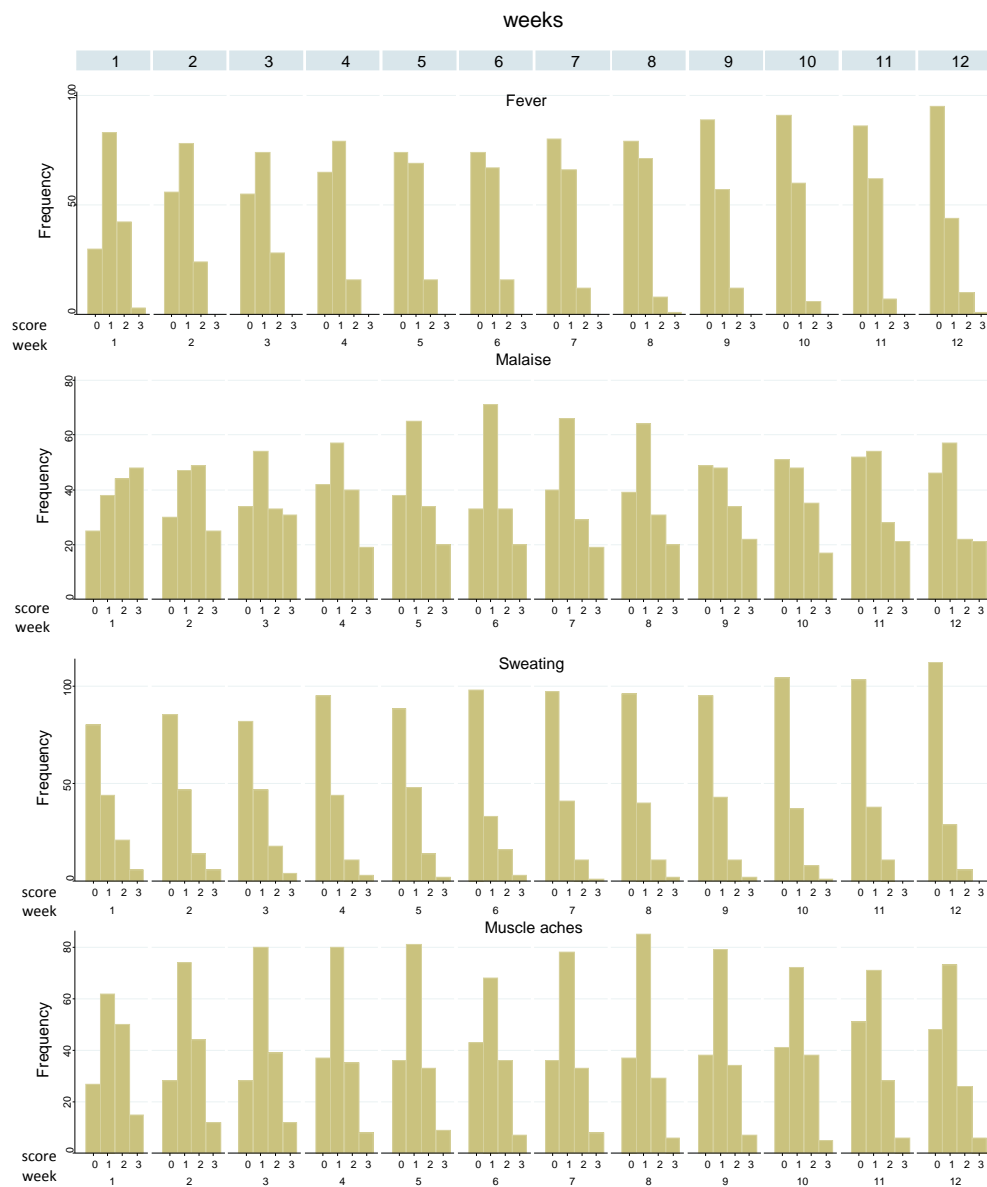

Supplement: S2 Fig — Histograms showing the change in symptoms’ score distribution over the follow-up. (PDF) [file pone.0135441.s003.pdf]

S3 Figure

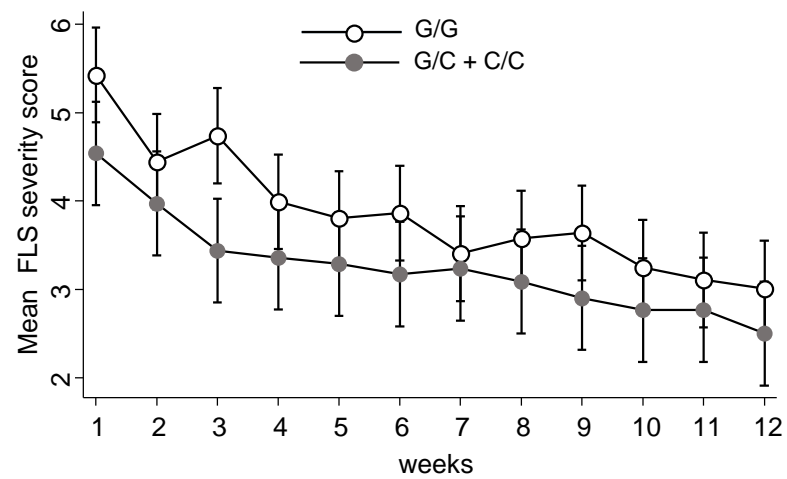

Supplement: S3 Fig — Graph represent the average FLS severity scores in MS patients divided according to the indicated IL-6 genotypes. (PDF) [file pone.0135441.s004.pdf]
