# Supplementary material for: Less Frequent and Less Severe Flu-Like Syndrome in Interferon Beta-1a Treated Multiple Sclerosis Patients with at Least One Allele Bearing the G>C Polymorphism at Position -174 of the IL-6 Promoter Gene
Source: PLoS One. 2015 Aug 18;10(8):e0135441. doi: 10.1371/journal.pone.0135441 (PMC4540473; doi:10.1371/journal.pone.0135441)
Supplement: S1 Table — (PDF) [file pone.0135441.s005.pdf]

S1 Table. Self-reported symptoms scoring as reported in patient diary.

Fever

Grade 0: no fever

Grade 1: fever between 37.1 and 38.0 °C

Grade 2: fever between 38.1 and 40.0 °C

Grade 3: fever higher than 40.0°C

Sweating

Grade 0: absent

Grade 1: mild (feeling of wet of skin)

Grade 2: important (feeling of wet clothes)

Grade 3: so important it may require cloth change

Muscle aches

Grade 0: absent

Grade 1: mild

Grade 2: moderate with reduced mobility

Grade 3: important with difficult mobility

Malaise

Grade 0: absent

Grade 1: mild and short lasting

Grade 2: moderate but short lasting (<6 hours)

Grade 3: important and longer lasting (>6 hours)
