# Supplementary material for: Less Frequent and Less Severe Flu-Like Syndrome in Interferon Beta-1a Treated Multiple Sclerosis Patients with at Least One Allele Bearing the G>C Polymorphism at Position -174 of the IL-6 Promoter Gene
Source: PLoS One. 2015 Aug 18;10(8):e0135441. doi: 10.1371/journal.pone.0135441 (PMC4540473; doi:10.1371/journal.pone.0135441)
Supplement: S2 Table — (PDF) [file pone.0135441.s006.pdf]

S2 Table. Results of the multivariable repeated-measures logistic regression used to estimate the probability of having FLS after each weekly injection.

Variable	Interpretation	OR	SE	significance
Week				
	1	reference category		
	2 vs. 1	0.419	0.217	
	3 vs. 1	0.144	0.075	***
	4 vs. 1	0.079	0.041	***
	5 vs. 1	0.138	0.071	***
	6 vs. 1	0.072	0.038	***
	7 vs. 1	0.080	0.042	***
	8 vs. 1	0.039	0.021	***
	9 vs. 1	0.037	0.020	***
	10 vs. 1	0.042	0.023	***
	11 vs. 1	0.049	0.026	***
	12 vs. 1	0.039	0.021	***
Peri-injection dose				
	without peri-injection dose	reference category		
	with vs. without peri injection dose	0.093	0.054	***
Week X peri-injection dose				
	(2 vs. 1) X (with vs. without peri injection dose)	1.289	0.923	
	(3 vs. 1) X (with vs. without peri injection dose)	4.823	3.509	*

		OR	SE	
	(4 vs. 1) X (with vs. without peri injection dose)	4.045	2.939	
	(5 vs. 1) X (with vs. without peri injection dose)	0.972	0.709	
	(6 vs. 1) X (with vs. without peri injection dose)	3.005	2.221	
	(7 vs. 1) X (with vs. without peri injection dose)	2.654	1.965	
	(8 vs. 1) X (with vs. without peri injection dose)	5.412	4.071	*
	(9 vs. 1) X (with vs. without peri injection dose)	7.388	5.539	**
	(10 vs. 1) X (with vs. without peri injection dose)	7.848	5.912	**
	(11 vs. 1) X (with vs. without peri injection dose)	3.218	2.409	
	(12 vs. 1) X (with vs. without peri injection dose)	4.237	3.258	
C allele				
	without C allele	reference category		
	with vs. without C allele	0.360	0.155	*
Total dose				
	0	reference category		
	1 vs. 0	4.651	1.550	***
	2 vs. 0	18.911	7.391	***
	3 vs. 0	45.536	22.365	***

N=145

OR: odds ratio; SE: standard errors

* p<0.05; ** p<0.01; *** p<0.001
